# Immune Escape Strategies in Head and Neck Cancer: Evade, Resist, Inhibit, Recruit

**DOI:** 10.3390/cancers16020312

**Published:** 2024-01-11

**Authors:** Kourtney L. Kostecki, Mari Iida, Bridget E. Crossman, Ravi Salgia, Paul M. Harari, Justine Y. Bruce, Deric L. Wheeler

**Affiliations:** 1 Department of Human Oncology, University of Wisconsin School of Medicine and Public Health, Madison, WI 53792, USA; kkostecki@wisc.edu (K.L.K.); iida@humonc.wisc.edu (M.I.); bridget.crossman@wisc.edu (B.E.C.);; 2 Department of Medical Oncology and Experimental Therapeutics, Comprehensive Cancer Center, City of Hope, Duarte, CA 91010, USA; rsalgia@coh.org; 3 University of Wisconsin Carbone Cancer Center, Madison, WI 53705, USA; jybruce@medicine.wisc.edu; 4 Department of Medicine, University of Wisconsin School of Medicine and Public Health, Madison, WI 53705, USA

**Keywords:** head and neck cancer, immune escape, immunotherapy, PDL1, cancer immunoediting

## Abstract

**Simple Summary:**

Head and neck cancer (HNC) is an aggressive form of cancer that affects hundreds of thousands of people worldwide and has a relatively poor prognosis. In the last decade, new therapeutics designed to enhance a patient’s immune system have been approved for use, but HNC has developed many different methods that help it escape the immune system. The existing immunotherapies target only one of these mechanisms, allowing HNC to utilize others to continue to elude the immune system. This review details the various strategies used by HNC to escape the immune response, dividing them into four general categories: evade, resist, inhibit, and recruit. Each of the immune escape mechanisms represents a potential immunotherapy target that could be used to treat HNC.

**Abstract:**

Head and neck cancers (HNCs) arise from the mucosal lining of the aerodigestive tract and are often associated with alcohol use, tobacco use, and/or human papillomavirus (HPV) infection. Over 600,000 new cases of HNC are diagnosed each year, making it the sixth most common cancer worldwide. Historically, treatments have included surgery, radiation, and chemotherapy, and while these treatments are still the backbone of current therapy, several immunotherapies have recently been approved by the Food and Drug Administration (FDA) for use in HNC. The role of the immune system in tumorigenesis and cancer progression has been explored since the early 20th century, eventually coalescing into the current three-phase model of cancer immunoediting. During each of the three phases—elimination, equilibrium, and escape—cancer cells develop and utilize multiple strategies to either reach or remain in the final phase, escape, at which point the tumor is able to grow and metastasize with little to no detrimental interference from the immune system. In this review, we summarize the many strategies used by HNC to escape the immune system, which include ways to evade immune detection, resist immune cell attacks, inhibit immune cell functions, and recruit pro-tumor immune cells.

## 1. Introduction

Head and neck cancer (HNC) is the sixth most common cancer worldwide, with approximately 66,500 new cases in the United States each year and over 16,000 associated deaths [1]. Often associated with alcohol use, tobacco use, and/or human papillomavirus (HPV) [2], HNC tumors arise from a variety of sites in the mucosal lining of the aerodigestive tract, such as the oral cavity, larynx, pharynx, and salivary glands. The prognosis for advanced stage HNC remains relatively poor, with five-year relative survival rates of less than 50% [1].

Clinical trials continue to refine optimal HNC treatment strategies, though only modest increases in overall survival have been achieved over the past decades. Early-stage HNC (stage I/II) can be effectively managed with surgery or radiation alone. However, the majority of HNC patients present with advanced disease (stage III/IV) for which multimodal treatment is required to achieve the highest probability of long-term survival.

In the 1990s–2000s, preclinical data from in vitro and in vivo studies [3,4,5] was validated in a landmark randomized trial using the epidermal growth factor receptor (EGFR)-targeting monoclonal antibody cetuximab. Combined with high-dose radiation, treatment with cetuximab increased survival for advanced-stage HNC patients over that achieved with radiation alone [6,7]. During the mid-2000s, improved understanding of significantly higher survival rates for HPV-associated HNC prompted efforts to reduce the toxicity burden in these patients. However, randomized trials (RTOG 1016 and De-ESCALaTE HPV) in p16-positive oropharynx cancer patients showed that radiation combined with cisplatin was superior to radiation combined with cetuximab with respect to both progression-free and overall survival [8,9]. Many other molecular targeting agents, including the anti-angiogenic agent bevacizumab and vascular endothelial growth factor receptor (VEGFR)2 inhibitors, showed potential in HNC when combined with radiation, but they have not altered the current clinical treatment landscape [10,11,12]. 

Immunotherapeutic agents came of age in the 2010s, when nivolumab and pembrolizumab demonstrated improved median survival rates in the metastatic/recurrent setting versus conventional chemotherapeutic agents, making a significant clinical impact in HNC [13,14,15]. Despite the hope that this benefit might be readily translated into the curative setting for HNC patients, the first major randomized trial using avelumab combined with chemoradiation (Javelin 100) showed no benefit of immunotherapy over chemoradiation alone in either progression-free or overall survival [16].

These early results emphasize the importance of investigating immune checkpoint inhibitors in the treatment of HNC and understanding how these agents interact with radiation and the immune system in the curative treatment setting. As noted, several immune checkpoint inhibitors (ICIs) have been approved by the Food and Drug Administration (FDA) for the treatment of HNC [13,17]. Unfortunately, only about 15% of HNC patients are initially responsive to ICI treatment [18], and those patients typically acquire resistance over time. 

Immune checkpoints were first discovered in the late 1980s, when Brunet et al. found cytotoxic T-lymphocyte-associated antigen 4 (CTLA4) in a screen of mouse cytotoxic T cell complementary deoxyribonucleic acid (cDNA) [19]. However, CTLA4’s function as a negative immune regulator was not understood until several years later [20,21]. Following closely behind, programmed cell death protein 1 (PD1) was discovered in 1992 [22], and its similar function as an inhibitory checkpoint was uncovered shortly thereafter [23,24]. In 1996, Leach and colleagues reported the first evidence of ICI efficacy, demonstrating that CTLA4 blockade had significant anti-tumor effect in murine cancer models [25]. Likewise, Dong et al. demonstrated efficacy of anti-PD1 therapeutics in the treatment of murine tumors in 2002 [26]. These studies and others eventually led to the first FDA approval of an ICI for cancer treatment—ipilimumab was approved for the treatment of melanoma in 2011 [27]. 

The field of immune checkpoints and ICIs is only 40 years old, but scientists have been attempting to harness the immune system of cancer patients for over a century [28]. While experiments with novel therapeutics have met with mixed success, the understanding of the immune system’s role in tumorigenesis and cancer progression has continually moved forward since the early 20th century. In 1908, Paul Ehrlich first posited that the immune system can and does inhibit the growth of cancer cells [29]. Decades of study followed, and in the 1950s, F. Macfarlane Burnet and Lewis Thomas contributed to what eventually became known as the cancer immunosurveillance hypothesis [30,31,32,33]. In this model, the immune system acts as a sentinel, constantly surveying the body for newly formed tumor cells and targeting any such cells for destruction [34]. While further study validated the immunosurveillance model [35], the early 2000s brought the recognition that immunosurveillance is only one part of the immune system’s interaction with cancer [36,37]. The umbrella model that arose as a result of these findings, called cancer immunoediting [36], is still utilized today to describe the relationship between the immune system and cancer. 

**Figure 1:**
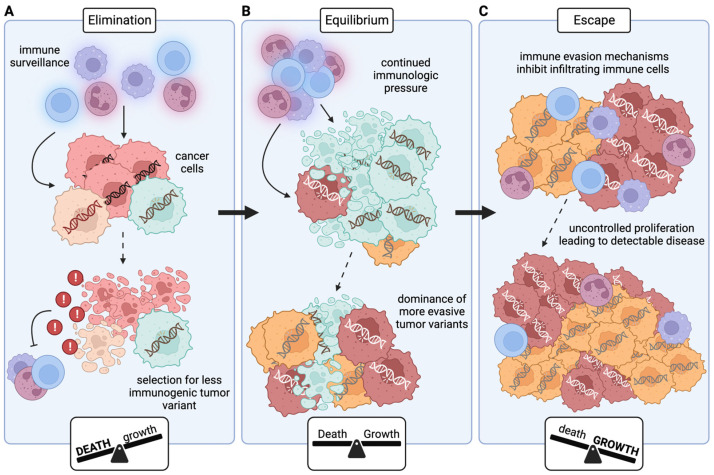
Three E’s of cancer immunoediting. (**A**) During the elimination phase, immune cells survey the body and kill any newly developed cancer cells; the rate of cancer cell death is much higher than the rate of cancer cell proliferation. However, the death of cancer cells can result in the release of molecules that inhibit immune cell function, and the immunologic pressure selects for tumor variants that employ one or more immune escape mechanisms. (**B**) The surviving, less immunogenic variants are able to proliferate at a sufficient rate to maintain their population, but their growth is kept in check by immune cells (or by a lack of nutrients resulting from insufficient vasculature). In the equilibrium phase, the rate of cancer cell death is approximately equal to the rate of cancer cell growth. Immunologic attacks again provide selection pressure, resulting in the dominance of even more immune evasive tumor variants. (**C**) The now-dominant immunosuppressive variants employ mechanisms to completely escape immune cells, allowing the cancer cells to proliferate uncontrollably and develop into clinically detectable tumors. The rate of cancer cell growth is much higher than the rate of cancer cell death in the escape phase.

Cancer immunoediting (Figure 1) consists of three phases: elimination, equilibrium, and escape [37]. The first phase, elimination, is what was previously referred to as cancer immunosurveillance, where effector cells continually survey the host and destroy any nascent cancer cells [38] (Figure 1A). However, the continual release of danger signals from dying cancer cells can eventually stimulate secretion of immunosuppressive molecules, hampering the ability of immune cells to eliminate cancer cells [39]. Further, the hallmark genetic instability of cancer cells [40] combined with high levels of host immune scrutiny results in selective pressure for less immunogenic tumor cells [41]. The decrease in immune cytotoxic potential mixed with decreased cancer immunogenicity can allow the cancer to move to the next phase: equilibrium (Figure 1B). In the equilibrium phase, the cancer is ‘dormant’, with the rate of cancer cell proliferation being matched by the rate of cancer cell death. This state can result from angiogenic dormancy, where the lack of vasculature slows proliferation [42], or from immune dormancy, where the rate of proliferation matches the rate of immune-mediated death [43]. Again, the genetic instability of cancer cells combined with continued immunologic pressure can result in selection for tumor variants that can elude the immune system, eventually leading to clinically detectable disease and complete immune escape, the final phase of cancer immunoediting (Figure 1C).

There are four major strategies used by cancer cells to overcome targeting by the immune system: evade, resist, inhibit, and recruit. Cancer cells can evade detection altogether, resist destruction by those immune cells they could not evade, inhibit those immune cells they could not resist, and/or recruit immune cells that create a pro-tumor environment [44]. In this review, we will summarize the combination of these four strategies used by HNC to escape the immune system.

## 2. Evade

Evading the immune system can be accomplished in a few ways. Cancer cells can prevent immune infiltration altogether through disruption of chemoattractant pathways, and, if that strategy fails, they can impair antigen-presentation (AP) machinery to escape detection.

Activation of the β-catenin/Wnt pathway has been shown to decrease levels of dendritic cell (DC) and T cell infiltration via downregulation of C-C motif chemokine ligand (CCL)4 [45]. In HNC, members of the Wnt pathway are highly overexpressed [46,47], and activation of the pathway has been correlated with lower levels of immune infiltrate [48]. Additionally, overexpression of hypoxia inducible factor (HIF)1α, HIF2α, and carbonic anhydrase 9 (CA9) is similarly associated with poor infiltration of immune cells [49]. Downregulation of the micro-ribonucleic acid (miRNA) 34a (miR-34a), associated with cell proliferation and angiogenesis in HNC [50], leads to decreased levels of pro-B cells, naïve cluster of differentiation (CD)8 T cells, and T helper (T_H_)1 cells [51]. 

Genomic aberrations can also impact immune infiltration. The phosphoinositide 3-kinase (PI3K) pathway has long been implicated in cancer progression [52], and mutations in PI3K pathway components are found in about a third of HNCs [53]. One downstream target of the PI3K pathway is phosphatase and tensin homolog (PTEN), loss of which results in increased expression of CCL2 and vascular endothelial growth factor (VEGF), which in turn can block T cell infiltration [54]. CUB And Sushi Multiple Domains 1 (CSMD1), another gene with a role in cancer progression, is lost in approximately 40% of HNCs; reduced or absent CSMD1 is associated with decreased T cell infiltration [55]. Tumor protein P53 (TP53) is one of the most commonly mutated genes in HNC, with approximately 70% of patients harboring one or more mutations [47,56]. Tumors with mutated TP53 have been shown to have decreased levels of B cells, CD8 T cells, and natural killer (NK) cells [57]. Finally, genomic cyclin-dependent kinase inhibitor 2A (CDKN2A) disruptions occur in nearly 80% of HNCs [56], and loss of heterozygosity in this gene is associated with lower levels of CD8 T cells, regulatory T cells (T_reg_s), and B cells [58].

To successfully evade detection by those immune cells that manage to infiltrate the tumor immune microenvironment (TIME), HNC cells can downregulate, mutate, or otherwise impair AP components [44,59,60]. Manipulation of these molecules is a delicate operation. NK cells will attack any cell lacking major histocompatibility complex class I (MHCI) expression, but having MHCI molecules puts tumor-associated antigens (TAAs)/tumor-specific antigens (TSAs) on display and leaves tumor cells vulnerable to T cell detection. 

Despite the risk of NK cell attack, some HNC cells downregulate MHCI directly [61]. This decrease in expression can sometimes result from genomic defects, including loss of heterozygosity or loss of allospecificity [56,61,62,63]. For example, loss of heterozygosity on chromosome 6, which encodes the heavy chains for MHCI, is found in ~40% of HNC tumors [62,64,65]. MHCI expression can also be affected by epigenetic changes [62]. Hypermethylation of CpG islands in MHCI promoter regions can result in expression loss; while minimal methylation of those regions can be detected in normal epithelial tissue, ~66% of HNC samples exhibit hypermethylation [66]. Yet another mechanism for MHCI downregulation is via the EGFR signaling pathway. EGFR, which is overexpressed in nearly 80% of HNCs [65], can alter the expression levels of MHCI by affecting the MHCI transcriptional regulator class II major histocompatibility complex transactivator (CIITA) [67]. Irrespective of cause, partial or total loss of MHCI expression has been detected in anywhere from 30 to 81% of HNCs [63,64,68,69,70,71], though most HNC cells express MHCI in sufficient quantities for antigen presentation, suggesting that immune evasion is likely achieved by alterations in other AP components [61,72,73]. 

The AP pathway involves many different molecules beyond MHCI itself, and loss of expression of even a single pathway component is sufficient to alter antigen presentation [65,74]. Low levels of transporter associated with antigen processing (TAP)1, TAP2, and tapasin are associated with lack of cytotoxic T lymphocyte (CTL) recognition [75], though they are certainly not the only AP components HNC downregulates to evade the immune system. Latent membrane protein (LMP)2 and LMP7 are commonly downregulated in HNC, as are beta-2-microglobulin (β_2_M), calnexin, calreticulin, and endoplasmic reticulum protein 57 (ERp57), though at varying frequencies (Table 1). Interestingly, while perturbation of any AP molecule can impair antigen presentation, Lopez-Albaitero et al. showed that reintroduction of TAP1 was sufficient to restore CTL recognition [73].

Expression of AP pathway components appears to be primarily regulated at the epigenetic and/or transcriptional level. In many cancers, processes like DNA methylation and histone hypoacetylation can decrease expression of AP molecules [62,76,77,78]. The methyltransferase enhancer of zeste homolog 2 (EZH2), part of a complex that catalyzes the methylation of histone 3 lysine 27 (H3K27), is associated with decreased AP component expression in HNC [79]. Zhou et al. found that EZH2 inhibition results in upregulation of AP molecules due to a reduction in the H3K27 methylation mark on the β_2_M promoter [80]. Transcriptionally, the interferon (IFN)γ-signal transducer and activator of transcription (STAT)1 pathway regulates AP component expression [81,82]. Lack of IFNγ or loss of IFNγ-response pathway components can result in downregulation or loss of AP molecule expression [74]. Some HNC cells harbor mutations in the IFNγ receptor gene [8], while others express decreased levels of phosphorylated STAT1 [81]. HNC cells may also overexpress Src homology-2 domain-containing protein tyrosine phosphatase (SHP)-2, which inhibits IFNγ-mediated STAT1 phosphorylation and therefore AP component expression [82]. Studies have shown that AP molecule downregulation can be overcome by exposure to IFNγ, such as that secreted by tumor-infiltrating immune cells, if the loss of AP component expression was not due to a genetic defect [65,70,73,75]. 

Finally, if tumor cells cannot prevent infiltration of or detection by immune cells, they can employ the evolutionary strategy of immunoediting. HNC tumors, on average, contain 2–6 distinct subclones [83], some of which may be more immunogenic than others. Clones with immunogenic epitopes are vulnerable to detection and killing by infiltrating immune cells, while clones without the now immune-dominant epitope escape unharmed and continue to proliferate [60,74,84]. However, few proteins that are overexpressed or mutated in HNC make for effective, immunogenic TAAs/TSAs able to trigger efficient immune responses [44,85]. Tumor-specific CTLs are often found in HNC patients independent of an effective immune response [84,85], suggesting that HNC tumors are generally unsuccessful in their attempts to evade the immune system entirely and therefore must be employing alternate immune escape strategies.

## 3. Resist

If cancer cells cannot evade immune detection, they may attempt to resist the immune system by preventing immune cell activation and/or preventing the immune cells from killing them. 

To prevent immune cell activation, HNC cells often downregulate the B7 family of molecules [86,87,88] via autocrine signaling with interleukin (IL)-6 and granulocyte-macrophage colony-stimulating factor (GMCSF) [89]. Loss of these B7 molecules, which normally provide a critical costimulatory signal to T cells during activation, can help prevent T cell-mediated cell death [44]. Cytokines can also stimulate immune activation, but the expression levels of pro-inflammatory T_H_1 cytokines are reduced in HNC [90,91], further promoting immune escape.

If cancer cells cannot prevent immune activation, they can attempt to prevent immune-mediated cell death via manipulation of apoptotic and cell cycle pathways. Downregulation of Fas [92] and CSMD1 [55], as well as overexpression of secreted phosphoprotein 1 (SPP1) [93,94], Toll-like receptor 4 (TLR4) [84,95], serine protease inhibitor (SERPIN) B1/4/9 [96,97], Fas associated phosphatase 1 (FAP-1) [98], FLICE-like inhibitory protein (c-FLIP) [99], decoy receptor (DcR)-1/2 [100,101], transient receptor potential cation channel (TRP)M2 [102], IL-2 [103], and IL-1α [104,105], has been associated with HNC resistance to apoptosis. 

Some of these molecules act by initiating or promoting survival signals. SPP1 activation resists apoptosis by triggering the mammalian target of rapamycin (mTOR)/PI3K/Akt and Janus kinase 2 (JAK2)/STAT3 [93,94] survival pathways, and TLR4 activation acts similarly via the PI3K/Akt and nuclear factor kappa B (NFκB) pathways [84,95]. TLR4 is activated by lipopolysaccharide (LPS), a component of Gram-negative bacterial cell walls, and, interestingly, HNC tumors are often found to be colonized by Gram-negative bacteria [95]. IL-1α overexpression induces activation of the activator protein 1 (AP-1) pathway, which in turn triggers B-cell lymphoma-2 (Bcl-2) expression and thus suppression of apoptosis [104,105]. 

HNC cells also perturb receptor-mediated apoptotic pathways. In normal head and neck tissues, expression of the death receptor Fas is found in ~85% of cells, but in HNC tissues, only ~1% of cells express Fas [92]. Without surface expression of Fas, immune cells expressing Fas ligand (FasL) cannot initiate Fas-mediated apoptosis. HNC cells also overexpress FAP-1 and c-FLIP to further protect against Fas-mediated apoptosis. FAP-1 binds directly to the negative regulatory domain of Fas itself and also offers protection via activation of the NFκB pathway [98], while c-FLIP acts as a decoy for caspase-8, resembling caspase-8 structurally but being unable to propagate the apoptotic signal [65,98]. Caspase-8 itself is mutated in 9% of HNCs [56]; these mutated versions remain constitutively bound to the adaptor molecule Fas-associated death domain protein (FADD) and thereby prevent proper recruitment of death-inducing signaling complex (DISC) components [106]. Also affected by caspase-8 mutations is the tumor necrosis factor (TNF)-related apoptosis-inducing ligand (TRAIL)-death receptor mediated apoptotic pathway, which is caspase-8 dependent. The TRAIL receptors themselves can also be altered, with polymorphisms in DR4 (TRAIL-R1) found in ~90% of HNCs [107]. Further, TRAIL decoy receptors DcR-1 and DcR-2 are overexpressed in HNC, providing another method of resistance to TRAIL-death receptor mediated apoptosis [100,101].

Other proteins work to directly counter immune-derived cytotoxins. SERPINs inactivate the effects of granzymes (molecules that trigger a caspase-independent apoptotic pathway) and are not typically expressed in normal head and neck tissues [96,97]. However, SERPINB9, which inactivates granzyme B, is found in 4% of HNCs; SERPINB4, which inactivates granzyme M, is found in 60% of HNCs; and SERPINB1, which inactivates granzyme H, is found in 30% of HNCs [97]. 

If immune-derived cytotoxins cannot be inactivated, HNC cells can alter the cell cycle to resist cytotoxic damage. HNCs often have mutations in proteins that regulate the G1/S cell cycle checkpoint, like p53, p21, and Rb [56,65], leading them to be more dependent on the G2/M cell cycle checkpoint for DNA repair [108]. This dependency gives cells more time to repair any damage sustained from immune cell attacks and helps prevent segregation of damaged DNA during mitosis [108]. Wee1 kinase has been found to be a key driver of G2/M cell cycle arrest in HNC, able to rescue cells from granzyme B-induced apoptosis [109,110,111].

## 4. Inhibit

While mechanisms for evading immune cell detection or resisting immune cell attacks can be effective, cancer cells can also inhibit infiltrating immune cells directly as another line of defense. HNC cells can inhibit immune cell activation/function, can disrupt immune cell maturation/differentiation, and can even induce immune cell apoptosis. 

HNC-derived exosomes containing cyclooxygenase-2 (COX2), transforming growth factor beta (TGFβ), PD1, and CTLA4 can promote CD8 T cell death and inhibit NK cell/CD4 T cell function [112,113]. Additionally, HNC cells expressing proteins like FasL [114,115,116,117], TRAIL [118], TNFα [118], galectin-1 [119], and programmed death ligand (PDL)1 [26,120] are able to induce apoptosis in tumor-infiltrating immune cells. Beyond direct immune cell killing, galectin-1 can prevent further T cell infiltration into the tumor via upregulation of galectin-9 and PDL1 [121]. 

PDL1 is overexpressed in 50–60% of HNCs [122,123], and expression leads to both immune cell apoptosis [26,120] and T cell dysfunction [124,125]. Extracellular factors, including HNC-derived exosomes [126] and cytokines (such as IFNγ, TNFα, GMCSF, and IL-4) [24,127,128,129], can induce PDL1 upregulation in HNC. Specifically, IFNγ regulates phosphorylation of STAT1/STAT3 through polycystic kidney disease 3 (PKD3), thus resulting in increased PDL1 expression [130,131,132]. In HNC, intracellular pathways also play a role in upregulating PDL1, including EGFR-mediated JAK2/STAT1 signaling [122] and the Axl/PI3K/Akt pathway [133,134]. Additionally, the cation channel TRPM8, which is overexpressed in HNC, increases PDL1 expression via the calcineurin-nuclear factor of activated T cells 3 (NFATc3) pathway [135]. The epithelial-to-mesenchymal transition (EMT) can also upregulate PDL1 [136]. The transcription factor zinc finger E-box binding homeobox 1 (ZEB1), activated during EMT, suppresses the miRNA miR-200, an inhibitor of PDL1 expression, thus allowing upregulation of PDL1 [137,138,139]. PDL2, an alternate ligand for PD1, is also expressed in HNC [140,141], where it plays a similar role in inhibiting T cell activation [142]. Post-translational modification, specifically glycosylation, enhances PDL1 and PDL2 activity via regulation of stability, translocation, and protein-protein interactions [143,144,145,146]. Stt3a is responsible for PDL1 glycosylation [147], while fucosyltransferase 8 (FUT8) glycosylates PDL2 [146]. As mentioned previously, the EGFR pathway is often aberrantly hyperactivated in HNC [65]; EGFR signaling via STAT3 can increase levels of FUT8 and thereby result in enhanced PDL2 activity [146]. Similarly, the cytokine TGFβ (overexpressed in HNC [148,149]) upregulates Stt3a expression via c-Jun activation, thus enhancing PDL1 activity [147]. 

Outside of the cancer cell, secreted TGFβ can directly affect immune cells such as DCs, T cells, and NK cells, suppressing maturation, activation, and/or cytotoxicity [150,151,152,153]. In NK cells, TGFβ reduces expression of activating receptor NK group 2 member D (NKG2D) and Fc receptor CD16 [154,155]. Levels of NKG2D can also be impacted by other HNC-derived cytokines. IL-6 and IL-8 are two cytokines that are highly overexpressed in HNC [90,156,157], and secreted IL-6/IL-8 can activate STAT3 in NK cells, resulting in downregulation of activating receptors NKG2D and NKp30 [158]. STAT3 signaling can also inhibit activation of T cells and DCs [159,160,161,162,163]. Increased STAT3 signaling can impair antigen-specific T cell responses, decreasing the amount of IL-2 secreted from the T cells and promoting a tolerogenic phenotype [159]. In DCs, STAT3 activation downregulates IL-12, MHC class II (MHCII), and CD40, impairing critical pro-inflammatory functions [161,162,163]. 

IL-6-mediated STAT3 activity in tumor cells can stimulate the release of factors, such as VEGF and IL-10, that inhibit DC maturation [150,159,163,164]. VEGF is overexpressed in most HNCs [165,166] and is known to inhibit DC maturation and antigen-presentation capabilities [44,59,61,167,168]. VEGF expression correlates with a reduced number of mature DCs and an increased number of immature DCs [169,170], possibly due to the inhibition of fms-like tyrosine kinase 3 (FLT3) ligand activity [171]. IL-10 has also been reported to interfere with DC maturation [163,172,173,174,175] and critical DC functions [172,175]. IL-10 decreases expression of costimulatory molecules [176], production of IL-12 [175,177], and antigen-presentation capabilities [175]. 

Another cytokine, IL-1α, is overexpressed in HNC and can induce the overexpression of IL-6 and C-X-C motif chemokine ligand (CXCL)8 in tumor cells; IL-6 and CXCL8 are known to inhibit macrophage functions [105,178,179,180]. HNC cells also overproduce the hormone prostaglandin E2 (PGE2) [90], which can interfere with monocyte functions such as migration and adherence to endothelial cells [181,182].

Membrane-associated gangliosides, produced by HNC cells and shed into the TIME [183], are another vehicle by which HNCs inhibit the immune system. These gangliosides inhibit the ability of monocytes and DCs to activate T cells by downregulating costimulatory molecules, MHC components, IL-12, and TNFα; they can also inhibit DC maturation and even DC differentiation from monocytes [184,185,186,187,188].

HNC cells secrete many different factors into the TIME to inhibit immune cells, but they also display various factors on their surface to achieve immune escape, including high mobility group box 1 protein (HMGB1) [189], carcinoembryonic antigen-related cell adhesion molecule 1 (CEACAM1) [190], CD47 [191], CTLA4 [192,193], and CD276 [194,195]. HMGB1 and CEACAM1, ligands for T cell immunoglobulin- and mucin-domain containing 3 (TIM3), bind to TIM3 on NK cells and cause downregulation of mixed lineage leukemia (MLL)T1, resulting in decreased expression of IFNγ/perforin and therefore impaired NK cytotoxicity [189,190,196]. CD47 is a ‘do not eat me’ signal that interacts with the protein signal regulatory protein alpha (SIRPα) on macrophages and DCs [197]. When CD47 binds to SIRPα, SHP-1 and/or SHP-2 are recruited to dephosphorylate motor protein myosin IIA, thus inhibiting the phagocytic process [198]. CTLA4 is a well-characterized immune checkpoint molecule; it competes with CD28 for binding to CD80/CD86 ligands on DCs [199]. CD28 binding to CD80/CD86 provides a critical costimulatory signal for T cell activation, but CTLA4 binding can prevent this process and thereby inhibit T cell activation [199,200]. Another immune checkpoint molecule, CD276 (B7-H3) has been found on the invasive front of HNC tumors, serving as a ‘shield’ against lymphocyte interference/infiltration by acting to suppress antigen-specific T cell activation and proliferation [194,195,201].

Finally, HNC cells can manipulate the surrounding environment itself, both directly and indirectly, to inhibit immune cell activation/function. Indirectly, HNC cells create regions of hypoxia with their rapid growth and abnormal angiogenesis [202,203], resulting in the secretion of adenosine and galectin-1, molecules known to be inhibitory to T cells [204,205,206,207]. Additionally, high rates of anaerobic glycolysis in HNC cells leads to the accumulation of extracellular lactate [74]. This lactate buildup inhibits the export of lactate from T cells and NK cells, which causes decreased IFNγ production and thus impairs cytotoxicity [208,209]. Further, extracellular lactate accumulation causes acidosis [210], which can lead to loss of T cell function [209,211]. HNC cells can also directly influence the environment through nutrient deprivation and production of inhibitory compounds. For example, HNC cells often overexpress glucose metabolism genes, thus depriving infiltrating T cells of the fuel needed for activation and expansion; overexpression of glucose transporter 1 has been correlated with decreased T cell infiltration in HNC [212,213]. Additionally, between 20 and 95% of HNCs overexpress the enzyme indoleamine 2,3-dioxygenase (IDO), which depletes the amino acid tryptophan [214,215]. Lack of available tryptophan causes T cells to arrest in the G1 phase of the cell cycle, and neither restoration of tryptophan nor costimulatory signaling through CD28 is able to restart cell cycle progression [216,217]. HNC cells also upregulate the enzymes CD39 and CD73, responsible for converting adenosine triphosphate (ATP) into adenosine [218,219,220,221], a molecule inhibitory towards T cells, DCs, and NK cells [222,223,224].

## 5. Recruit

**Figure 2:**
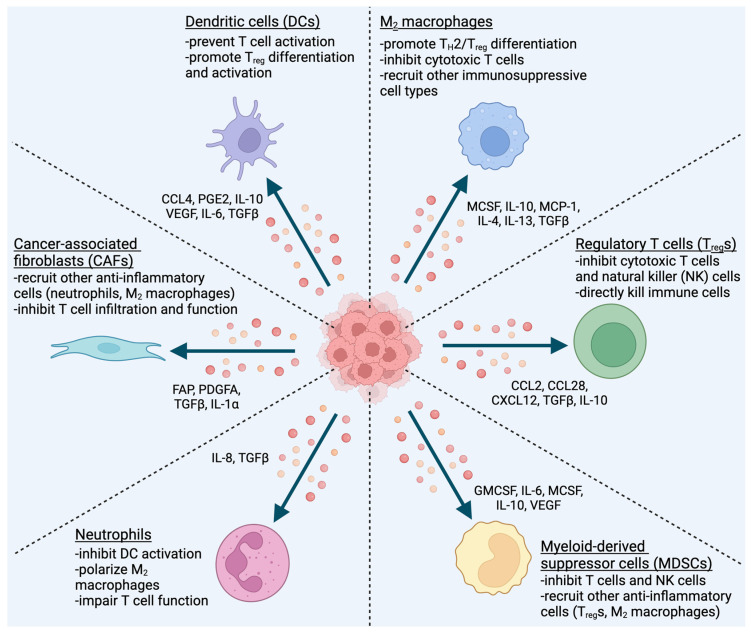
HNC cells secrete cytokines, chemokines, and other molecules to recruit immunosuppressive, pro-tumor cell types. The soluble factors released by cancer cells can also polarize, activate, and/or differentiate the recruited cells to enhance their anti-inflammatory effects.

Cancer cells employ a variety of methods for evading, resisting, or inhibiting immune cells. These strategies for immune escape all involve direct interaction between the cancer cell (or a cancer cell-derived product) and the immune system. The final strategy for immune escape instead relies on the recruitment of intermediary cells, which then inhibit immune cells and create a pro-tumor environment. HNC cells recruit many different cell types to serve as these intermediaries, including DCs, macrophages, T_reg_s, myeloid-derived suppressor cells (MDSCs), neutrophils, and cancer-associated fibroblasts (CAFs) (Figure 2). 

### 5.1. Dendritic Cells (DCs)

Conventional DCs are recruited to the tumor by CCL4, CCL5, and X-C motif chemokine ligand (XCL)1 [225]; CCL5 and XCL1 are typically produced by NK cells [225], while CCL4 is produced by tumor cells [45]. Once at the tumor, HNC-derived cytokines manipulate DC function and maturation to create an immunosuppressive environment. HNC-derived PGE2 [90,226] and IL-10 [90,91,227] decrease the amount of IL-12 produced by DCs, which then results in an increase in T_H_2 differentiation [228]. The loss of IL-12 also inhibits proper antigen-presentation in DCs, thus preventing DC-mediated T cell activation [175,229]. In addition to HNC-derived IL-10, DCs can themselves be induced by HNC cells to secrete IL-10, thereby resulting in IL-12 loss, increased T_H_2 differentiation, and inhibition of T cell activation [175,228,229]. 

As discussed above, HNC cells overexpress IL-6 [156] and VEGF [165,166], suppressing DC maturation via STAT3 activation [230,231] and FLT3 ligand inhibition [171], respectively. These immature DCs express low levels of MHCII and the costimulatory molecules CD80/CD86, thereby impairing antigen presentation and preventing T cell activation [167,169,232,233]. Further, HNC-derived IL-10, VEGF, and TGFβ convert immature DCs into tolerogenic DCs, which can induce T cell tolerance by promoting the activation and differentiation of T_reg_s [175,233,234,235,236,237,238,239]. 

Besides conventional DCs, HNC cells also recruit plasmacytoid DCs (pDCs) via CXCL12 and CXCL14 overexpression [240,241,242]. While some pDCs are anti-tumor [243] (typically in HPV + HNC [244]), the majority are pro-tumor [131,243], having reduced expression of IFNα and costimulatory molecules [242] and stimulating the recruitment of T_reg_s [241].

### 5.2. Macrophages

Macrophages are another immune cell type recruited by HNC to promote an immunosuppressive TIME. Many cytokines and chemokines have been implicated in the recruitment of macrophages to the TIME, including CCL3/4/5/8 [245,246,247], VEGF [245,246,247,248], macrophage colony stimulating factor (MCSF) [245,246,247,248], IL-10 [248], platelet-derived growth factor (PDGF) [248], CCL18/20 [249], CXCL12 [249], TGFβ [155,250,251], and monocyte chemoattractant protein 1 (MCP-1) [250,251,252,253]. Tumor-associated macrophages (TAMs) as a whole are linked to poor clinical outcomes in HNC [254], though TAMs are generally divided into two subtypes, M1 and M2, that each have distinct biological characteristics [255]. Activated M1 macrophages stimulate a T_H_1-like, pro-inflammatory immune response, while M2 macrophages stimulate a T_H_2-like, anti-inflammatory response [256,257]. Typically, the majority of TAMs are M2s [258], and these tumor-infiltrating M2 macrophages have been associated with a poor prognosis in HNC patients [259,260,261]. 

M2 polarization is induced by cells in the TIME secreting cytokines and other factors, including IL-4 and IL-13 [262,263,264,265,266,267], MCSF [268,269], IL-10 and TGFβ [270,271], and IL-8 [262,272]. HNC cells overexpress T_H_2 cytokines IL-14, IL-13, IL-10, and TGFβ [89,91,227,273,274], and HNC stromal cells also produce significantly increased levels of TGFβ [275,276]. Environmental conditions in the tumor can further stimulate M2 polarization: production of IL-10, TGFβ, and MCSF is increased under hypoxic conditions [270,271,277,278], and the acidosis caused by high rates of anerobic glycolysis in tumor cells [74] can enhance M2 differentiation [279]. Additionally, macrophage expression of C-C motif chemokine receptor (CCR)2 and IL-4Rα is essential for M2 polarization and survival [280,281,282]. Macrophages can also be polarized to the M2 state following engulfment of apoptotic tumor cells [283]. In the non-cancer setting, this transition promotes wound-healing and tissue regeneration, but in tumors, it instead fosters an immunosuppressive TIME [278,283].

M2 macrophage polarization results in the expression and secretion of factors that create an immunosuppressive environment, stimulating T_H_2 and T_reg_ differentiation and inhibiting cytotoxic T cells, NK cells, and DCs [245,246,284,285,286]. M2 macrophages upregulate TGFβ, PGE2, IL-10, arginase 1 (ARG1), peroxisome proliferation activated receptor gamma (PPARγ), IL-1Rα, VEGF, TNFα, IL-1, IL-6, IL-8, and GMCSF, all of which contribute to immune suppression [245,246,247,287,288,289,290,291,292,293]. 

Secretion of TGFβ plays a major role in promoting an immunosuppressive environment by (1) inducing T_reg_ differentiation/activation [152,153,278,294,295]; (2) inhibiting the differentiation, maturation, activation, and proliferation of CTLs [278,291,295]; (3) inhibiting the maturation and function of DCs [237,296]; (4) inhibiting M1 macrophages [287,290]; and (5) recruiting (and polarizing) other immunosuppressive cells, such as CAFs [153], M2 macrophages [250,251,270,271], and N2 neutrophils [297,298,299,300]. Similarly, IL-10 production results in (1) induction of T_reg_ differentiation/activation [301,302], (2) inhibition of DC maturation and function [175,238,296], and (3) recruitment of MDSCs [303,304]. In HNC, secretion of TGFβ and IL-10 are associated with reduced survival time [259]. Further, upregulation of ARG1 contributes to T cell dysfunction by depleting the TIME of arginine, a required metabolite for T cell proliferation [291,305,306]. Lack of arginine prevents T cells from utilizing oxidative phosphorylation, thus decreasing survival capacity and anti-tumor activity [307,308,309]. 

M2 macrophages also express CD39 and CD73, ectonucleotidases that convert ATP into adenosine [218,278,310,311,312,313]. As mentioned previously, adenosine is an immunosuppressive molecule, inhibiting NK cells, T cells, and DCs [222,223,224]. More specifically, adenosine inhibits the antigen presentation function of DCs and other antigen-presenting cells (APCs), thereby preventing T cell activation [314]. Adenosine also promotes the proliferation and activation of M2 macrophages and T_reg_s, thus contributing to an immunosuppressive TIME [222,223,315]. 

Another way M2 macrophages promote immune suppression and escape is through the production of reactive oxygen species (ROS) and reactive nitrogen species (RNS) [305,306,316]. High levels of ROS can negatively impact T cell proliferation and activation [317], as well as promote differentiation of anti-inflammatory T_H_2 cells [318,319]. Macrophages also release RNS after infiltrating the tumor, resulting in nitration of CCL2 and thereby preventing CCL2-mediated recruitment of CTLs [316]. 

Finally, M2 macrophages express immune checkpoint ligands like PDL1, PDL2, and CTLA4, which suppress T cell responses [305,306,320,321]. HNC cells induce PDL1 expression in macrophages via an IL-10-dependent process [322], while PDL2 overexpression is mediated by the CCL2/CCR2 pathway [282]. As discussed previously, PDL1/PDL2 signaling results in T cell dysfunction [124,125,142] and can induce apoptosis in immune cells [26,120], and CTLA4 impairs T cell activation through competition with the critical costimulatory molecule CD28 [199,200].

### 5.3. Regulatory T Cells (T_reg_s)

In addition to macrophages and DCs, HNC cells also recruit T_reg_s to help themselves escape the immune system [323,324]. T_reg_s are an extremely immunosuppressive cell type that supports tumor progression through the inhibition of NK cells, DCs, B cells, and even other T cells [325,326]. They can be recruited either as ‘natural’ T_reg_s or as naïve CD4 T cells that are polarized into T_reg_s once inside the tumor [327,328]. Those T_reg_s that are polarized in the tumor are more immunosuppressive than ‘natural’, circulating T_reg_s, typically expressing higher levels of immune checkpoint molecules [329]. HNC cells recruit T_reg_s through several chemokines and their associated receptors, including CCL2/CCR4, CCL28/CCR10, and CXCL12/C-X-C motif chemokine receptor (CXCR)4 [330,331,332,333]. 

Once T_reg_s reach the tumor, differentiation/polarization and activation is induced by a variety of tumor- and immune-derived factors, including TGFβ [113,152,153,294], IL-10 [301,302], PGE2/COX2 [112,113,155], IL-35 [301,302], adenosine [222,223,315], IL-6/STAT3 [334], and even has_circ_0069313, a tumor-derived circular RNA that helps T_reg_s maintain FoxP3 levels [335]. Further, T_reg_s function through the expression of immunosuppressive cytokines and immunomodulatory receptors, including TGFβ, IL-10, IL-35, and other T_H_2 cytokines [296,323,324,336,337,338], as well as CD39/CD73 [256,313,339,340] and immune checkpoint molecules like CTLA4, TIM3, lymphocyte activation gene 3 (LAG3), and PD1 [329,341,342]. 

As described above, TGFβ and IL-10 have immunosuppressive effects on the TIME, where they inhibit polarization/activation/maturation/function of M1 macrophages [287,290], DCs [175,237,238,296], and CTLs [153,295,323,324,336] and recruit/activate/polarize M2 macrophages [250,251,270,271], MDSCs [303,304], N2 neutrophils [297,298,299,300], CAFs [153], and more T_reg_s [152,294,301,302]. Similarly, IL-35 functions to suppress T cell proliferation and activity while also promoting the differentiation/function of more T_reg_s [343,344,345,346]. 

Just like M2 macrophages, T_reg_s express CD39 and CD73, enzymes responsible for metabolizing ATP and generating adenosine [218,311]. As mentioned above, adenosine has immunosuppressive effects on the TIME, inhibiting the functions of NK cells, M1 macrophages, T cells, and DCs [224,314,340,347] and promoting the proliferation/activation of M2 macrophages and T_reg_s [222,223,315].

CTLA4 and PD1, as discussed above, function to inhibit T cell responses. CTLA4 binds to CD80/CD86 on APCs, thus preventing interaction with the T cell costimulatory molecule CD28 [340]. Further, the binding of CTLA4 to CD80/CD86 on DCs promotes the generation of tolerogenic IDO+ DCs [233,348]; IDO depletes the TIME of tryptophan, which irreversibly causes T cells to arrest in the G1 phase of the cell cycle [214,216,217]. Likewise, LAG3 promotes immune tolerance via suppression of CTL recruitment and activity [341,342], and TIM3 expression promotes T_reg_ function and CTL inhibition [349,350,351]. 

T_reg_s can also directly kill other immune cells; through the secretion of granzyme B and perforin, they can kill NK cells and T cells [337,338,352]. In addition, T_reg_s can induce Fas-mediated apoptosis in CD8 T cells [353].

### 5.4. Myeloid-Derived Suppressor Cells (MDSCs)

Another cell type, MDSCs, are recruited by HNC to promote an immunosuppressive TIME and thus escape the immune system. HNC cells produce GMCSF [89,90,91], IL-6 [89,90,91,180], MCSF [354,355], IL-10 [90,91,301,302], VEGF [90,165,166], PGE2/COX2 [182,226,356,357], IDO [214,215], MCP-1 [250,251], and IL-8 [90,180], all of which play a role in MDSC recruitment [303,358,359]. The hypoxic conditions typically found in tumors can also recruit MDSCs via the interaction of the HIF-1α/macrophage migration inhibitory factor (MIF) and NFκB/IL-6 axes [303,358,359] as well as the stimulation of MCSF production [277,278]. Though MDSCs are naturally anti-inflammatory, they can be differentiated further to an even more immunosuppressive phenotype by MCSF [360,361,362] and PGE2 [363]. 

MDSCs create an immunosuppressive TIME through the secretion of IL-10 [364,365,366], TGFβ [278,295], PGE2/COX2 [155,367], and MCSF [368,369], as well as the expression of ARG1 [370,371,372,373], IDO [374], PDL1 [375,376], and inducible nitric oxide synthase (iNOS) [370,373,377,378]. The secretion of IL-10 and TGFβ results in recruitment/activation of T_reg_s [278,295,332,379], while M2 macrophages are recruited and polarized by the release of IL-10 and MCSF [368,369,380]. Similarly, MDSC-derived PGE2/COX2 can induce T_reg_ production [155] as well as induce STAT1/STAT3 phosphorylation [367], which in turn results in the upregulation of ARG1 [309] and IDO [374]. 

As discussed above, the enzyme IDO is responsible for metabolizing tryptophan, a critical nutrient for T cell function [214,215]. In addition to simply depleting tryptophan, IDO also converts tryptophan into immunosuppressive kynurenine metabolites [374]. Cysteine, another nutrient critical for T cell activation and function, is similarly depleted from the local environment by MDSC-mediated sequestration [381]. 

MDSC expression of ARG1 and iNOS inhibits T cell and NK cell responses, both through nutrient depletion and through production of immunosuppressive molecules [370,373,377,382]. ARG1 and iNOS both metabolize arginine, leading to the production of ornithine and nitric oxide (NO), respectively, as well as simply depleting arginine from the local environment [364,383,384]. Depletion of arginine from the TIME results in T cell inhibition, as arginine is essential for correct CD3 expression [385]. Additionally, arginine deprivation results in T cells being shunted away from oxidative phosphorylation, decreasing survival capacity through the inhibition of cell cycle regulators cyclin D3 and CDK4 [307,308,309,386].

Nitric oxide inhibits the immune response through a variety of mechanisms. Production of NO inhibits MHCII expression, thus preventing antigen presentation and activation of T cells [387]. Further, NO impairs Fc receptor binding on NK cells, reducing cytotoxicity and impairing signal transduction [388]. Nitric oxide also inhibits T cells and NK cells by rendering cells unresponsive to IL-2 via nitrosylation of cysteine residues on proteins in IL-2 response pathways [389]. Lack of response to IL-2 inhibits T cell proliferation/activation [390] and NK cell activation/cytotoxic potential [391]. 

Beyond direct effects on the immune system, NO can react further with superoxide to produce peroxynitrite, an immunosuppressive RNS molecule [392]. Peroxynitrite can induce apoptosis [393] or anergy [394] in activated T cells, and it can also cause nitration of the TCR/CD8 complex, altering the specific peptide binding and thus rendering T cells unresponsive to antigen-specific stimulation [394,395].

### 5.5. Neutrophils

Neutrophils are another immune cell type associated with HNC progression and immune escape. In HNC patients, high neutrophil infiltration is positively associated with tumor stage and negatively associated with overall survival [396]. Neutrophils are recruited by HNC-derived IL-8 [397] and are polarized to an immunosuppressive, N2 phenotype by TGFβ [298,299,300]. Tumor-associated neutrophils (TANs) can inhibit T cell responses through expression of ARG1, PDL1, IL-10, and ROS [371,372,398,399], and TANs can actually induce CD8 T cell apoptosis through NO production and the TNFα pathway [400]. Additionally, TANs can polarize macrophages to the M2 phenotype through MCSF production [368,369]. TANs also produce CCL4 and CCL5, which recruit DCs to the TIME [401], as well as myeloperoxidase and elastase, which inhibit DC activation [402].

### 5.6. Cancer-Associated Fibroblasts (CAFs)

Finally, HNC cells recruit CAFs [194], which, while not immune cells, contribute to the development of an immunosuppressive TIME through their effects on immune cells such as macrophages, T cells, MDSCs, and DCs [403]. In HNC, CAFs are strongly correlated with invasion, recurrence, treatment resistance, and ultimately poor patient outcomes [404,405]. 

HNC expression of fibroblast-associated protein (FAP) [406], platelet-derived growth factor A (PDGFA) [407], and TGFβ [275,276] all contribute to the recruitment of CAFs [153,408,409], while HNC-derived IL-1α promotes CAF proliferation [410]. Once they reach the TIME, CAFs regulate an immunosuppressive environment via the secretion of cytokines, chemokines, and growth factors, such as VEGF, epidermal growth factor (EGF), IL-6, TGFβ, TNF, and CXCL8 [276,411,412,413], though there is some evidence suggesting that the immunosuppressive effects of CAFs are mediated through FAP and CXCL12 [414,415]. Neutrophils and macrophages can be recruited by CAF-derived CXCL12 [298,416], while T cells can be excluded from the tumor in a CXCL12-dependent manner [414,415].

CAFs can then induce M2 macrophage and N2 neutrophil polarization both through direct secretion of TGFβ [275,276] and through secretion of cardiotrophin-like cytokine factor 1 (CLCF1), which can induce expression of TGFβ in tumor cells [417]. Further, CAF-derived IL-6 activates STAT3 in neutrophils, resulting in upregulation of PDL1 and thus T cell inhibition [417,418]. 

T cells are also affected by CAFs, through direct and indirect mechanisms. In HNC, CAFs inhibit T cell proliferation via the PDL1/PDL2 axis and can even induce effector T cell apoptosis [413]. Additionally, CAF-derived CCR4 and IL-1β induce CCL22 overexpression in HNC cells, thus promoting T_reg_ recruitment and activation [413,419].

## 6. Conclusions and Future Directions

Head and neck cancer is a major global health issue, impacting hundreds of thousands of lives each year. The classic treatments of surgery, radiation, and chemotherapy have been recently joined by immunotherapies, though only a small subset of patients respond to these new therapeutics [18]. Understanding the mechanisms of primary and acquired resistance to ICIs is therefore an area of extensive research [420,421,422,423]. Of note, however, is that the only two ICIs approved for use in HNC target the same immune checkpoint, PD1/PDL1. 

In this review, we discussed some of the many mechanisms by which HNC can escape the immune system. HNC cells can evade immune detection altogether, resist attacks from immune cells, inhibit immune cells directly, or recruit immunosuppressive, pro-tumor immune cells. While the PD1/PDL1 immune checkpoint is one of these mechanisms, it is certainly not the only mechanism utilized by HNC to escape the immune system. Each of the discussed immune escape strategies represents a potential immunotherapeutic target that could be used to improve treatment outcomes in HNC.

## Figures and Tables

**Table 1:** Antigen-processing (AP) components are downregulated with varying frequency in head and neck cancer (HNC). Studies on AP component expression alterations were performed on primary HNC samples and/or on HNC cell lines.

AP Component	HNCs That Downregulate	References
TAP1	34–71%	[64,68,69,70,71]
TAP2	64–88%	[64,68,70]
Tapasin	40–79%	[64,68,69,71]
LMP2	78–88%	[64,70,71]
LMP7	~60%	[64,70]
β_2_M	60–87%	[64,71]
Calnexin	~34%	[71]
Calreticulin	~19%	[71]
ERp57	~26%	[71]

## Data Availability

Not applicable.
